# Share pledge and accounting conservatism in share-pledging firms: Evidence from a natural experiment in China

**DOI:** 10.1371/journal.pone.0306899

**Published:** 2024-07-09

**Authors:** Xin Wang, Yue Sun, Yanlin Li, Cuijiao Zhang

**Affiliations:** 1 School of Accounting, Southwestern University of Finance and Economics, Chengdu, Sichuan, China; 2 School of International Business, Southwestern University of Finance and Economics, Chengdu, Sichuan, China; 3 School of Economics and Management, Sichuan Tourism University, Chengdu, Sichuan, China; 4 School of Business and Law, Edith Cowan University, Joondalup, Australia; Ho Chi Minh City Open University, VIET NAM

## Abstract

This paper focuses on firms in which insiders pledge their shares as collateral for loans. By investigating a natural experiment—China’s enactment of provisions on share reductions that restrict pledge creditors’ cashing-out behavior—we find that pledging firms exhibited more conservative financial reporting after the implementation than non-pledging firms. This effect was pronounced in firms with a higher ratio of pledged shares, a longer maturation period of the pledged shares, and more concentrated pledge creditors. Additionally, we show that pledging firms increased their accounting conservatism after the shock, leading to a lower risk of margin calls and stock price crashes. The effect on accounting conservatism was stronger in firms with controlling pledgers or when the pledge creditors were banks. Our results remained consistent after we performed several robustness tests. These behaviors are economically logical because the provisions heighten creditors’ liquidity risk and the potential losses of loan default. Pledging shareholders embrace more accounting conservatism to mitigate creditors’ concerns about agency costs and avoid triggering margin calls. Our findings provide direct support that creditors have a real demand for accounting conservatism and highlight the impact of shareholder-creditor conflicts on the financial reporting policies of pledging firms.

## 1. Introduction

In cross-regional financial markets, certain key shareholders in listed firms tend to pledge their holding shares to creditors for debt financing. Studies document that share pledging exacerbates information opacity [1, 2]: Pledging shareholders have an incentive to manipulate earnings [3, 4], make optimistic forecasts [5, 6] and suppress negative news [7] to maintain control rights during the pledging period. While such opportunistic behaviors benefit pledging shareholders by boosting stock prices, the effects tend to be short-lived [8]. Earnings management is likely to be detected by creditors eventually [9]. Additionally, a firm’s attempts to conceal mounting negative news raises the likelihood of a stock price crash, which may trigger margin calls and undermine firm value [7, 10, 11]. The potential damage caused by share pledging has prompted scholars and regulators to consider ways to stabilize this form of debt financing. However, there is a gap in the literature on how share pledging can positively impact accounting information disclosure. Prior literature has explored how other forms of debt financing can improve financial reporting conservatism [12–15]: Debtors enhance accounting conservatism to mitigate creditors’ concerns about agency costs [16, 17]. In that vein, our paper investigates whether pledging shareholders have an incentive to embrace greater accounting conservatism and mitigate pledge creditors’ concerns about loan risk.

To this end, we conducted a natural experiment made possible by a policy reform enacted by the Chinese government. In 2017, the China Securities Regulatory Commission (CSRC) enacted “Several Provisions on the Reduction of Shares Held in a Listed Company” (the Provisions), which block various channels of share reduction for major shareholders of Chinese-listed firms (i.e., shareholders who hold more than 5% of the total equity). Additionally, the Provisions apply to pledged shares, which increases the liquidity risk to pledge creditors when they want to sell pledged shares. Prior to the Provisions, pledge creditors could sell the pledged shares when the stock price fell to the liquidation line in the pledge, which reduced creditors’ agency costs and avoided loan default risk [11, 18, 19]. Due to the new rules constraining share reductions, creditors will be highly concerned about agency costs arising from debtors’ risk-shifting actions, which benefits shareholders at the expense of creditors [20, 21]. Compared with non-pledging firms, pledging firms have a greater incentive to release low-risk signals to creditors and resolve shareholder-creditor conflicts. Otherwise, pledge creditors may take protective action before the default occurs, such as requiring shareholders to make supplementary pledges as collateral. Although the Provisions affect all companies with major shareholders, we can use the generalized differences-in-differences model to test the treatment intensity between pledging and non-pledging firms following the enactment of the Provisions [22, 23].

In this paper, we advance that accounting conservatism is an effective way for pledgers to release low-risk signals to pledge creditors and alleviate pledge creditors’ concern about agency costs. Pledgers’ conservatism serves to convince pledge creditors that the former will persist in risk avoidance after debt financing is obtained [16, 24]. Specifically, firms with high accounting conservatism are less likely to engage in activities with operational risks [25, 26]. Furthermore, conservative financial reports alleviate agency problems between shareholders and creditors [14, 16, 27] and mitigate information asymmetry [28], thereby lowering creditors’ collateral requirements [29].

Our results show that pledging firms exhibited more conservative reporting after (vs. before) the implementation of the Provisions; this effect was pronounced in firms with higher pledged share ratios, a longer maturation period of pledged shares, and more concentrated pledge creditors. Moreover, we found that the improvement of accounting conservatism was associated with fewer margin calls and a lower risk of stock price crash. These results suggest that improving accounting conservatism is an effective way to reduce creditors’ proactive actions. Additionally, we show that the increase in accounting conservatism was more significant when the pledgers were controlling shareholders or when the pledge creditors were banks. These findings indicate that controlling pledgers have a stronger incentive to increase accounting conservatism than other pledging shareholders, while banks have more power to pressure pledging shareholders than other creditors. Our main findings remained consistent even after we used alternative measures of accounting conservatism and performed several robustness tests.

We acknowledge that the Provisions apply to both creditors and pledging shareholders. The enhanced accounting conservatism may simply be due to pledging shareholders’ eagerness to sell shares without creditors’ effect. To alleviate that concern, one of our robustness tests excluded the sample in which either executives or shareholders sold their shares. Our baseline results were unchanged.

Our study contributes to the literature in several ways. First, our estimates indicate that share pledge improves the accounting conservatism of listed firms subject to the Provisions. Prior studies have mainly focused on the negative impact of insiders’ personal share pledge behaviors on corporate financial reports, such as earnings management, optimistic forecasts and the withholding of negative news [3–7]. By demonstrating that major shareholders’ share pledges increase corporate accounting conservatism, we shed light on a positive aspect of share pledging: namely, improving the quality of financial reports. Our findings differ from Xu (2021), who asserted that pledging shareholders decreased conservatism and resorted to overly optimistic financial reports to prevent minority shareholders from discovering their expropriation behaviors [6]. By contrast, we highlight that the Provisions prevent major shareholders’ expropriation behaviors and prompt pledging shareholders to enhance accounting conservatism.

Second, our estimates show that pledge creditors influence the behavior of pledging shareholders. The prior literature has explored the behavior of pledging insiders, focusing on the wedge between ownership and control rights, as well as the control transfer. Due to the separation of ownership and control rights, pledging shareholders engage in expropriation at the expense of minority shareholders [30–32]. In order to maintain their control rights, controlling shareholders adopt opportunistic measures after share pledging to elevate stock prices and avoid forced sales [33–35]. However, the literature has yet to explore whether pledge creditors play a role in influencing the behavior of pledging shareholders. By analyzing the shock of a sudden restriction on the reduction of pledged shares, this paper demonstrates that the Provisions heightened pledge creditors’ liquidity risk and concerns about agency costs. Consequently, pledging shareholders enhance accounting conservatism to mitigate creditors’ concern and avoid creditors taking proactive actions.

Third, our paper contributes to the literature on how regulation affects pledging firms’ reporting quality. Porta et al. (1998) were the first to investigate the influence of legal rules on investor protection [36]. Since then, scholars have explored the impact of legal systems on firm value [37], self-dealing [38], controlling shareholders’ tunneling behavior [39] and firm innovation [40]. While share pledging is an increasingly common financing channel worldwide, corporate scandals associated with the activity have led to growing concern from regulators, investors, and scholars. Our results underscore that regulation positively impacts pledging firms’ reporting quality, effectively alleviating stakeholders’ concerns about share pledging. By studying the effectiveness of China’s regulatory strategies on corporate governance, other countries can gain valuable insights and adopt similar approaches to address their own governance challenges.

The remainder of this paper is organized as follows: In Section 2, we introduce the institutional background of the Provisions in China, develop our hypotheses, and discuss the related literature. In Section 3, we describe our data sources, perform the descriptive analyses, and construct the empirical models. In Section 4, we present our empirical results. In Section 5, we perform several additional tests. In Section 6, we perform multiple robustness tests. In Section 7, we present our conclusions.

## 2. Institutional background, hypothesis development, and related literature

### 2.1. Share reduction provisions of 2017

On May 27, 2017, the CSRC promulgated the Provisions, which mainly apply to large shareholders holding more than 5% of a firm’s outstanding shares. The Provisions contain several stipulations: First, shares sold through call auctions within 90 consecutive days must not exceed 1% of the firm’s total outstanding shares, while block trades should not surpass 2% of the total outstanding shares. Second, transferrers and transferees must continue to abide by this reduction ratio for another six months after the transaction, through the method of agreement transfer, if the reduction behavior leads the shareholder to lose its major shareholder status. Third, the shares held by major shareholders and persons acting in concert must be calculated together. These restrictions on share reduction behavior are intended to ensure financial stability and protect investors’ rights.

The Provisions also apply to pledge creditors who can short pledged shares. In the past, pledged shares could be liquidated through block transactions and agreement transfers. Pledge creditors could sell the pledged shares on the capital market when the stock price fell to the liquidation line of pledge, which reduced creditors’ agency costs under loan default risk [11, 18, 19, 41]. In other words, creditors were not obligated to pay close attention to the governance of pledging firms. The Provisions completely blocked these reduction channels, which increase creditors’ liquidity risk and potential losses from loan defaults.

**Fig 1:**
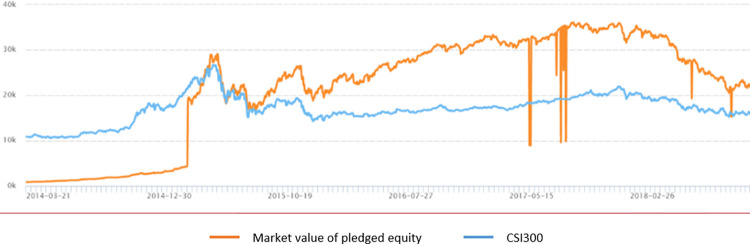
Reaction of the equity pledge market to the implementation of the provisions.

The stock pledge market reacted strongly to the implementation of the Provisions. We present a graph of the changes in the market value of pledged equity in Fig 1. After the implementation of the Provisions on May 27, 2017, the market value of pledged equity dropped significantly. Notably, the CSI 300 index remained stable, which indicates that the decline of the new pledged equity was not caused by changes in the stock market index, but by the introduction of the Provisions.

### 2.2. Related literature and hypothesis development

Researchers have shown significant interest in the economic consequences of share pledging. First, share pledging intensifies the conflict between controlling and minority shareholders. After pledging the shares, controlling shareholders maintain control rights over the pledged stocks, but their corresponding cash flow rights are temporarily immobilized. This misalignment between the control and cash flow rights exacerbates agency problems between controlling and minority shareholders [42, 43]. Controlling shareholders engaging in share pledging are motivated to divert firm resources at the expense of minority shareholders, leading to inferior performance and a decline in firm value [31, 32]. Second, shareholders who engage in share pledging face significant stock price pressure due to margin calls and forced sales. Typically, under the terms of a loan agreement, if stock prices fall below the liquidation line, a margin call is triggered that requires pledging shareholders to provide additional collateral. Failure to meet the required margin empowers lenders to sell their pledged shares to close out the associated loans and prevent default loss. This forced sale can lead to the loss of firm control for controlling shareholders. To maintain their control rights, controlling shareholders may adopt opportunistic measures to elevate stock prices, including share repurchases [33, 34], reductions in future innovation activities [44, 45] and excessive financialization behavior [35]. Share pledging also has a negative impact on firms’ reporting quality: Insiders who pledge shares are incentivized to conceal negative news, make positive financing reports or manipulate earnings to elevate stock prices [3, 4, 6, 7]. Hu et al. (2021) argued that, when facing the threat of losing control rights, controlling shareholders even collude with analysts to make optimistic forecasts [5].

However, the previous strategies that boost stock prices may not last in the long run, as the stock price may reverse in subsequent periods [8, 9]. The continuous withholding of accumulated bad news is unsustainable. All negative information surfaces in the market beyond a certain threshold, leading to a significant decline in stock prices (commonly referred to as a stock price crash), which may trigger loan default and the forced sales of pledged shares [7, 10]. These opportunistic activities undermine both corporate governance and future performance [11, 32].

The Provisions increase pledge creditors’ concern about agency costs and may influence the behavior of pledging shareholders. The Provisions increase pledge creditors’ liquidity risk when there is a demand to realize a large number of pledged shares within a short period. Prior to the Provisions, pledge creditors could avoid agency costs by selling pledged shares when the stock price fell to the liquidation line in pledge. Following the enactment of the Provisions, pledge creditors can no longer rely on short selling to reduce the cost of debt and protect their interests, as the value of pledged shares realized by creditors fails to cover their debt loss in the event of loan default [46]. In this case, the creditors will be highly concerned about agency costs arising from debtors’ risk-shifting actions, which benefit shareholders at the expense of creditors [20, 21]. Common examples include increasing leverage via cash payouts (dividends or share repurchases) and raising the riskiness of assets through various investments. To alleviate creditors’ concerns, pledging shareholders are incentivized to release low-risk signals to creditors and resolve shareholder-creditor conflicts [47, 48]. Otherwise, pledge creditors may require pledging shareholders to make supplementary pledges to boost collateral before a default occurs. Meeting additional margin calls can be difficult for an insider, since the initial pledge decision is generally due to insufficient liquidity [11]. Additionally, triggering margin calls releases negative signals to the market, potentially leading to a decline in stock price [5, 44].

One effective strategy that pledging shareholders can use to reassure creditors of their commitment to risk avoidance, and thereby mitigate concerns about agency costs, is accounting conservatism. This orientation toward financial reporting has three benefits. First, pledge creditors require financial reporting to assess whether shareholders might take high-risk actions that do not align with creditors’ best interests [13]. Financial reporting is well suited for providing pledge creditors with information regarding net assets, leverage, current-period performance, near-term cash flows, changes in asset riskiness, etc. Conservative financial reporting offers accurate financial data and leads to decreases in information asymmetries, thus satisfying the information needs of external stakeholders [49]. Accounting conservatism appears to be an effective signal of firms’ low operational risk [24, 26, 50]: Highly conservative firms are less likely to engage in money laundering activities [25] and more vigilant about managing risks associated with contractions in credit supply [26]. Thus, accounting conservatism can serve as a strategy employed by pledgers to address conflicts between shareholders and creditors by committing to hedge risk [16]. Second, conservative reporting affects creditors’ lending decisions: Pledging firms with high accounting conservatism can provide reliable accounting information, which helps to mitigate the information asymmetry between listed firms and external stakeholders, thereby improving credit and reducing the collateral requirements from pledge creditors [28, 29, 49]. Pledge creditors are more willing to offer the debt at a lower price when they are less concerned about agency costs [48, 51]. Moreover, creditors offer lower interest rates to conservative debtors, whose reports offer lenders a more timely signal of default risk [52]. Moreover, accounting conservatism limits managers’ motivation and capability to exaggerate performance or conceal unfavorable news to investors, which, in turn, reduces stock price crash risk [14, 53, 54]. As the leading concern of pledging shareholders is to prevent additional margin calls after the Provisions, without facing the downward risk of the stock price, they may pursue conservative financial reports. Therefore, we propose our first hypothesis as follows:

*Hypothesis 1*: *Following the enactment of the Provisions*, *pledging shareholders increased the accounting conservatism of pledging firms*.

There are several factors that can affect the influence of pledging on accounting conservatism. The first is the pledge ratio. In listed firms, debt tends to incentivize shareholders to prioritize their interests over those of creditors, increasing the agency cost of debt and the likelihood of loan default [20, 55]. The debt ratio is positively associated with creditors’ relative bargaining power to transfer the firm’s control rights [15]. In the case of share pledges, the larger the share pledge ratio, the more shareholders may be concerned about margin calls, which further enhance the risk of control transfer [33, 56]. Faced with that risk, shareholders may improve corporate governance and alleviate agency conflicts between debtors and creditors [57, 58]. As a component of corporate governance, more conservative financial reporting helps firms obtain better debt ratings and reduce the cost of debt [15, 27]. By placing limits on share reduction, the Provisions exacerbate pledge creditors’ liquidity risk and concerns about agency costs. When the proportion of pledged shares is higher, pledge creditors have higher power to trigger margin calls before a default occurs. Therefore, shareholders with higher pledged share ratios have a stronger incentive than other shareholders to improve accounting conservatism, mitigating creditors’ concern about agency costs. On that basis, we propose the following hypothesis:

*Hypothesis 2*: *After the enactment of the Provisions*, *firms with a higher ratio of pledged shares exhibited a higher level of accounting conservatism*.

The second factor is the length of the remaining pledge period. A long maturity period is associated with a risk premium that may reflect uncertainties about debtors’ ability and willingness to repay [59, 60]. Therefore, a long maturity period increases pledge creditors’ concerns about agency costs [61] and may be associated with greater uncertainty about the firm’s business [15, 62]. As the firm’s business volatility increases, signals of a covenant violation become less apparent, increasing creditors’ demand for conservative financial information [15]. Due to the Provisions, pledge creditors have an increased incentive to ask pledging shareholders to supplement pledged shares or repay in advance. In this situation, debtors are motivated to increase their accounting conservatism in order to release risk avoidance signals to pledge creditors. On this basis, we hypothesize:

*Hypothesis 3*: *Following the enactment of the Provisions*, *firms with a longer period to the maturity of pledged shares exhibited a higher level of accounting conservatism than firms with a shorter period to the maturity of pledged shares*.

The third factor is the concentration of pledge creditors. Concentrated creditors have better access to information about the firm’s quality and risk factors [63, 64]. This information advantage motivates concentrated creditors to invest more in monitoring and influencing borrowers’ financial reporting choices [65]. Additionally, concentrated creditors have more bargaining power than less concentrated creditors to accelerate payments and require margin calls [66, 67]. Faced with that bargaining power, debtors will exhibit more conservative financial reporting to satisfy creditors [15]. Therefore, it is possible that the conservatism effect is more pronounced when creditors are concentrated. In this setting, pledging shareholders are motivated to increase their accounting conservatism to avoid a margin call; after all, concentrated pledge creditors have greater power to threaten pledging shareholders with supplemental share pledges or repayments in advance. Accordingly, we predict:

*Hypothesis 4*: *Following the enactment of the Provisions*, *firms with more concentrated pledge creditors exhibited a higher level of accounting conservatism than firms with less concentrated pledge creditors*.

## 3. Research design

### 3.1. Sample

Our sample consists of listed firms on the Shanghai and Shenzhen stock exchanges from 2014 to 2019. China’s reform of the new stock issuance system (implemented in November 2013) and the revised “Securities Law of the People’s Republic of China” (enacted on March 1, 2020) imposed strict requirements for information disclosure. Both regulations created harsh penalties for companies involved in false reporting, misleading statements, or material omissions. We restricted the sample period to 2014–2019 to mitigate the influence of external policies on the dependent variable and obtain accurate empirical results. We took the following steps to select the sample: (1) excluding special treatment firms (identified as those suffering financial distress) and firms that went public in or after 2017; (2) excluding firms in the financial and insurance industries; (3) excluding observations with missing variables, and (4) winsorizing the variables at the 1% and 99% levels to reduce the influence of outliers. The final raw sample contained 14,873 firm-year-level observations. We reduced this sample to 12,848 observations after performing propensity score matching (PSM). The financial data came from the China Stock Market and Accounting Research (CSMAR) database and Chinese Research Data Services Platform (CNRDS). The data on stock price returns came from NetEase Finance. NetEase Finance is one of China’s renowned internet financial information service platforms. The data on pledged shares came from the Choice database. Choice database is a financial data analysis and investment management software developed by Shanghai Oriental Wealth Financial Data Services Co., Ltd.

### 3.2. Model construction

To test Hypothesis 1, we followed Goodman (2018) and Schmidheiny and Siegloch (2023) by constructing generalized differences-in-differences model (1) [22, 23]. This choice is made because, although the provisions affect all companies with major shareholders, the treatment intensity is higher in pledging firms than in non-pledging firms. The model is as follows:

$${C\_Score}_{i,t}/{Cons}_{i,t}=\beta +\gamma {Pledge}_{i}\times {Post}_{t}+r{Control}_{i,t}+u_{i}+u_{t}{+\varepsilon }_{i,t}$$ (1)

where the dependent variable is either *C_Score* or *Cons*, which proxy the accounting conservatism in listed firms [24, 68]. We also used alternative measures of accounting conservatism in one of the robustness tests [69]. We mainly focused on the coefficient of the interaction, which indicates the real effect of the Provisions.

Next, we constructed *Pledge*, which was equal to 1 when any major shareholder held more than 5% of the total shares as of the enforcement date of the Provisions, and 0 otherwise. This is because the Provisions mainly increased the liquidity risk for major shareholders’ creditors. We used the PSM method to match observations in the treatment (*Pledge* = 1) and control (*Pledge* = 0) groups, then adopted the matched sample for difference-in-differences (DID) analysis. The time dimension variable, *Post*, was 1 if the observation was in 2018 or later, and 0 otherwise.

In addition, we followed related studies [27, 28, 70–74] by controlling for *Size*, *Lev*, the growth rate of operating income (*Growth*), the net cash flow from operating activities divided by total assets (*CFO*), common dividends divided by the market value of common stock (*Div_Yield*), *MB*, whether the company has been sued (*Litigation*), a state-owned enterprise dummy (*SOE*), the age of the listed company (*Firm_Age*), capital expenditures divided by total assets (*Capex*), the fraction of independent directors (*Outside_Dir*), and board size (*Board_Size*). We used the Big 4 as a proxy of external audit quality (*Big4*), which is known to enhance financial information quality [50], Since the separation of ownership and control gives rise to agency problems between managers and shareholders—another potential source of the demand for conservatism [75]—we also controlled the percentage of the firm’s shares held by the CEO at the end of the fiscal year (*Mas*). Additionally, we controlled for the time fixed effect (*u*_*t*_) and firm fixed effect (*u*_*i*_).

Following Nunn and Qian (2011) [76], we tested Hypotheses 2 to 4 by designing models (2) to (4), respectively, as follows:

$${C\_Score}_{i,t}/{Cons}_{i,t}=\beta +\gamma {P\_Ratio\_Variables}_{i}\times {Post}_{t}+r{Control}_{i,t}+u_{i}+u_{t}{+\varepsilon }_{i,t}$$ (2)

$${C\_Score}_{i,t}/{Cons}_{i,t}=\beta +\gamma {P\_Time\_Variables}_{i}\times {Post}_{t}+r{Control}_{i,t}+u_{i}+u_{t}{+\varepsilon }_{i,t}$$ (3)

$${C\_Score}_{i,t}/{Cons}_{i,t}=\beta +\gamma {CN}_{i}\times {Post}_{t}+r{Control}_{i,t}+u_{i}+u_{t}{+\varepsilon }_{i,t}$$ (4)

In model (2), we used two variables to measure the proportion of pledged shares (*P_Ratio_Variables*): *P_Ratio*, which is the average ratio of pledged shares to the total shares owned by majority shareholders in a treatment group firm when the Provisions were implemented and *maxP_Ratio*, which is the largest ratio of accumulated shares pledged by any majority shareholder to the total shares in a treatment group firm in the year when the regulations were implemented. The coefficients of the interactions reflect the real change in accounting conservatism in pledging firms caused by the proportion of pledged shares after the implementation of the Provisions. In model (3), we used two variables to proxy the remaining pledge time (*P_Time_Variables*): *P_Time*, which indicates the average remaining years of majority shareholders’ pledges in a treatment group when the Provisions were enforced, and *longP_Time*, which is the largest number of remaining pledging years among majority shareholders’ pledges for a treatment group firm in the year that the Provisions were implemented. In model (4), following Ongena et al. (2012) [77], we define *CN* as the concentration of pledge creditors, which can be computationally expressed by model (5):

$${CN}_{i,t}=\sum_{j=1}^{n}S_{ij}^{2}$$ (5)

where *S* indicates the ratio of the pledged shares of one creditor to the total equity, *i* represents the listed firm in the treatment group and *j* represents the creditor. *CN* is measured using the Herfindahl–Hirschman Index (HHI) of the proportion of majority shareholders’ pledged shares to the total equity for each creditor. The coefficient of the interaction in model (4) reflects the impact of pledged creditor concentration on accounting conservatism in pledging firms after the implementation of the Provisions.

### 3.3. Descriptive statistics

The descriptive results are provided in Panel A of Table 1. Both *C_Score* and *Cons* proxy accounting conservatism. The mean and median of *C_Score* are 0.06 and 0.02, respectively, and those of *Cons* are 0.07 and 0.04, respectively. In terms of the control variables, the mean values of *SOE*, *Size*, and *Lev* are 0.34, 22.31, and 0.42, respectively. Panel B reports the differences in accounting conservatism (*C_Score* and *Cons*) before and after the implementation of the Provisions. In the treatment group, the mean value of *C_Score* after the implementation is 0.119, which is 0.104 more than the mean before the implementation, representing a significant increase in accounting conservatism. In the control group, the increase in *C_Score* after the implementation is 0.096, which is smaller than the increase in the treatment group. The trend is consistent when the variable used is *Cons*.

**Table 1:** Descriptive statistics.

***Panel A*: *Descriptive analysis***						
**Variable**	***N***	**Mean**	**S.D.**	**P25th**	**Median**	**P75th**
**C_Score**	14,873	0.06	0.08	0.01	0.02	0.09
**Cons**	14,873	0.07	0.08	0.02	0.04	0.10
**Pledge**	14,873	0.57	0.50	0.00	1.00	1.00
**Post**	14,873	0.42	0.49	0.00	0.00	1.00
**P_Ratio**	14,873	0.02	0.03	0.00	0.01	0.03
**maxP_Ratio**	14,873	0.05	0.07	0.00	0.03	0.09
**P_Time**	14,873	0.73	0.84	0.00	0.49	1.29
**LongP_Time**	14,873	1.19	1.31	0.00	0.69	2.38
**CN**	14,873	0.01	0.02	0.00	0.00	0.01
**Size**	14,873	22.31	1.29	21.39	22.14	23.05
**Lev**	14,873	0.42	0.20	0.26	0.41	0.57
**Growth**	14,873	0.41	1.03	-0.02	0.15	0.46
**CFO**	14,873	0.05	0.07	0.01	0.05	0.09
**Div_Yield**	14,873	0.01	0.01	0.00	0.00	0.01
**Big4**	14,873	0.06	0.24	0.00	0.00	0.00
**MB**	14,873	2.08	1.33	1.25	1.66	2.40
**Litigation**	14,873	0.11	0.32	0.00	0.00	0.00
**SOE**	14,873	0.34	0.47	0.00	0.00	1.00
**Firm_Age**	14,873	2.02	0.92	1.39	2.08	2.83
**Capex**	14,873	0.05	0.04	0.01	0.03	0.06
**Outside_Dir**	14,873	0.38	0.05	0.33	0.36	0.43
**Board_Size**	14,873	8.50	1.68	7.00	9.00	9.00
**Mas**	14,873	0.10	0.16	0.00	0.00	0.17
***Panel B*: *Univariate analysis***						
**Variable**			**Mean** **(After)**	**Mean** **(Before)**	**Difference** **(After–Before)**	**T-value**
**C_Score**	**Treatment**		0.119	0.015	0.104***	76.775
	**Control**		0.113	0.017	0.096***	59.749
**Cons**	**Treatment**		0.137	0.028	0.109***	73.854
	**Control**		0.129	0.033	0.096***	55.239

Note: Panel A provides descriptive statistics for the variables. Panel B presents the results of a comparison of the dependent variables before and after the implementation of the Provisions in the treatment and control groups. P25th and P75th denote the 25th and 75th percentiles, respectively. See the S1 Appendix for variable definitions.

### 3.4. Propensity score matching and parallel trend test

Following Aghamolla and Li (2018), we used the PSM method with nearest-neighbor and 1:1 matching without replacement to avoid sample selection bias [78]. In terms of matching variables, we followed Dou et al. (2019) in selecting *Growth*, *CFO*, *Firm_Age*, *Capex*, *Outside_Dir*, and *Board_Size* as the control variables [11]. Additionally, we controlled for year and industry fixed effects. As shown in Table 2, of the 12,848 observations that remained after the matching were equally split between the treatment and control groups. For most of the variables, there were no significant differences between the treatment and control groups.

**Table 2:** Difference test of matching variables.

Panel A: Comparison of sample characteristics							Panel B: Logit regression
	Pre-match			Post-match			Pre-match
	Treatment group	Control group	Difference	Treatment group	Control group	Difference	(1)
	(1)	(2)	(3)	(4)	(5)	(6)	
**Growth**	0.420	0.386	0.034**	0.378	0.386	-0.008	0.007
			(2.00)			(0.43)	(0.40)
**CFO**	0.043	0.054	-0.011***	0.0540	0.054	0.000	-2.126***
			(-9.45)			(-0.06)	(-8.46)
**Firm_Age**	1.963	2.092	–0.129***	2.072	2.092	-0.020	-0.038*
			(–8.46)			(-1.25)	(-1.87)
**Capex**	0.046	0.044	0.002***	0.041	0.044	-0.003***	2.130***
			(2.97)			(-3.68)	(4.83)
**Outside_Dir**	0.379	0.374	0.005***	0.372	0.374	-0.002*	-0.957**
			(5.13)			(-1.95)	(-2.53)
**Board_Size**	8.320	8.739	-0.419***	8.638	8.740	-0.102***	-0.137***
			(-15.17)			(-3.50)	(-10.81)
**Constant**							2.222***
							(8.13)
**Year fixed effect**							Yes
**Industry fixed effect**							Yes
**N**							14,873
**adj. R2**							0.05

Note: Panel A shows the differences in matching variables between the treatment group and the control group. Panel B reports the regression results of the first stage of PSM. See the S1 Appendix for variable definitions.

**Fig 2:**
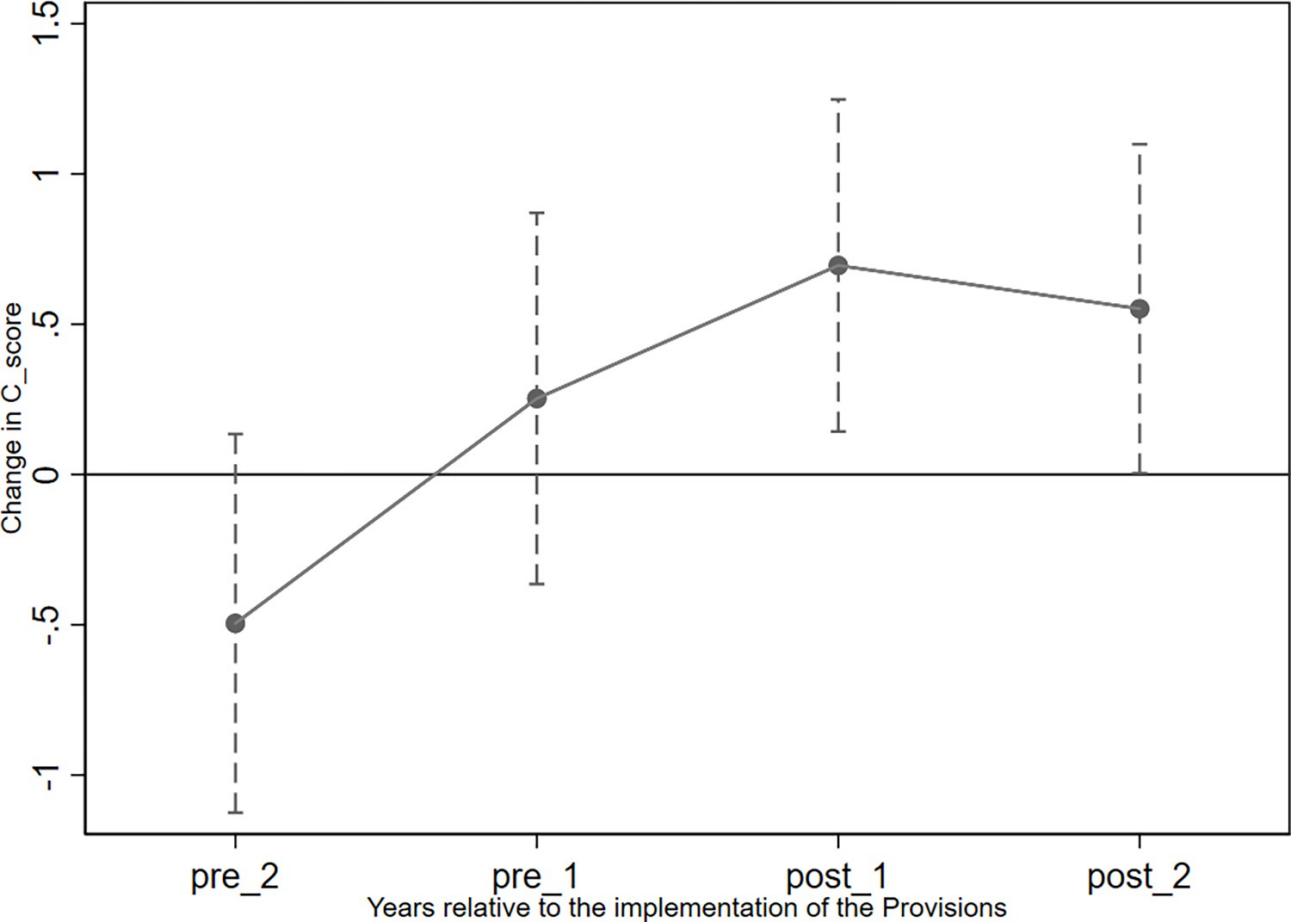
Parallel trend test for the variable *C_Score*.

**Fig 3:**
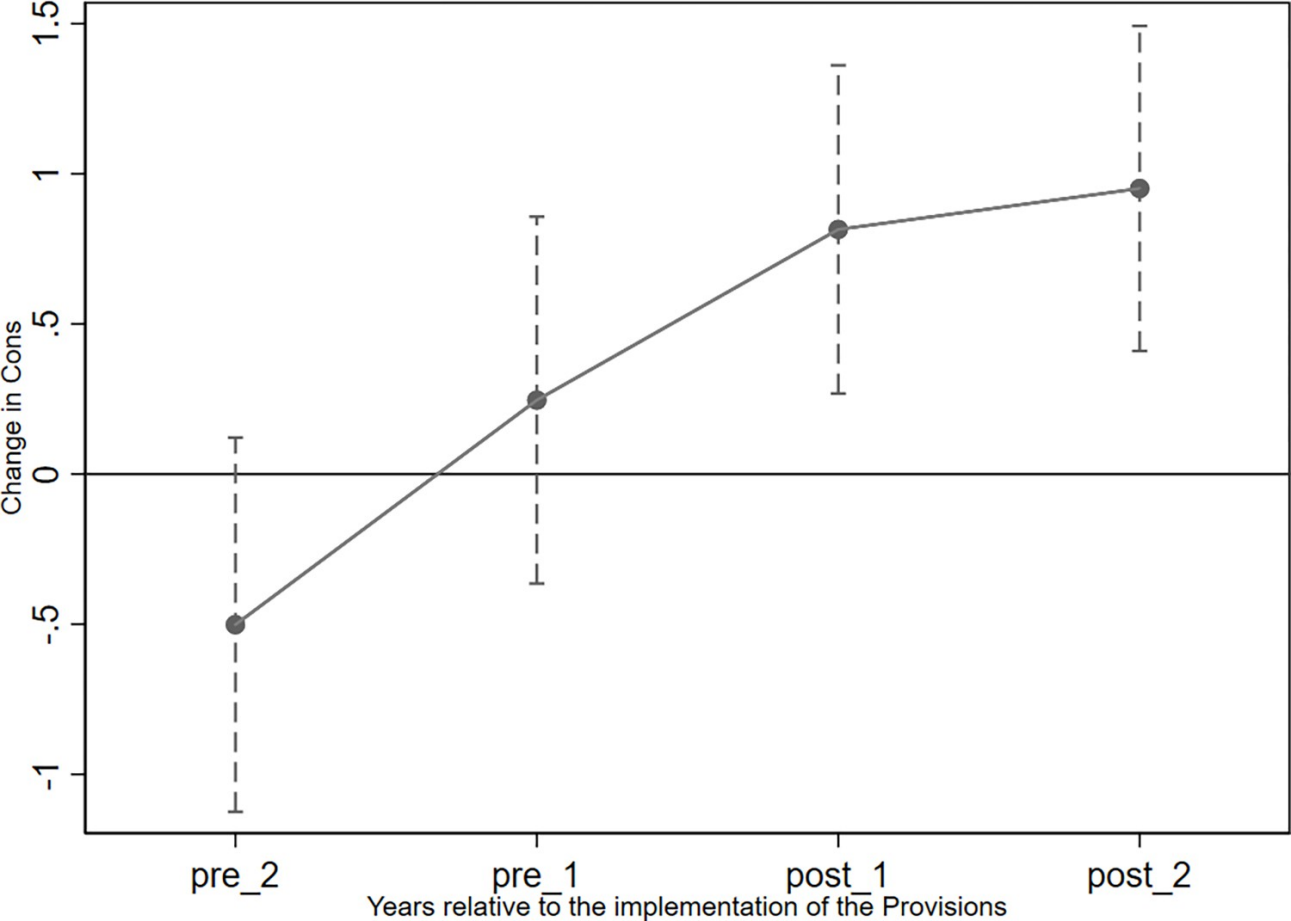
Parallel trend test for the variable *Cons*.

The parallel trend assumption, namely that changes in *C_Score* and *Cons* in the treatment and control groups before the shock of the implementation of the Provisions are indistinguishable, should be supported by our data. To reveal the trend of accounting conservatism in pledging firms (treatment group) and non-pledging firms (control group) before and after 2018, we followed Beck et al. (2010) by replacing *Post* in model 5 with the year dummy variables *pre_2*, *pre_1*, *post_1*, and *post_2—*which equaled 1 in 2015, 2016, 2018, and 2019, respectively, and 0 otherwise*—*and performing the regression [79]. Figs 2 and 3 show the coefficients of the interactions between the year dummy variables and *Pledge* before and after the shock, alongside their 95% confidence intervals. We find that the 95% confidence intervals of the coefficients in 2015 (*pre_2*) and 2016 (*pre_1*) included 0, indicating that these coefficients were not significantly different from 0 in these years; thus, our PSM-DID analyses satisfy the parallel trend assumption. However, the 95% confidence intervals of *post_1* and *post_2* were significantly larger than 0.

## 4. Regression analysis

### 4.1. Share pledge and accounting conservatism in pledging firms

Table 3 reports the change in the accounting conservatism of pledging firms after the implementation of the Provisions. The coefficient of *Pledge×Post* is 0.005 and 0.007 in columns 1 and 2, respectively, and both values are significant. We obtained similar results with *Cons* as the dependent variable. These results show that the accounting conservatism of pledging firms significantly increased following the implementation of the Provisions. Therefore, the empirical evidence supports Hypothesis 1: The Provisions increased creditors’ liquidity risk and their concerns about agency costs, which inclined pledging shareholders to adopt greater accounting conservatism to convey low-risk signals to their creditors.

**Table 3:** Share pledge and accounting conservatism in pledging firms.

Variable	*C*_*score*		*Cons*	
	(1)	(2)	(3)	(4)
**Pledge×Post**	0.005**	0.007***	0.010***	0.009***
	(2.50)	(3.20)	(4.48)	(4.52)
**Size**		0.004*		0.002
		(1.65)		(0.74)
**Lev**		0.131***		0.153***
		(17.55)		(20.76)
**Growth**		-0.000		0.000
		(-0.12)		(0.02)
**CFO**		0.037***		0.035***
		(3.76)		(3.56)
**Div_Yield**		0.189***		0.139**
		(2.80)		(2.08)
**Big4**		0.003		0.004
		(0.50)		(0.67)
**MB**		-0.006***		-0.009***
		(-8.07)		(-11.49)
**Litigation**		0.007***		0.007***
		(3.68)		(3.52)
**SOE**		0.004		0.002
		(0.69)		(0.36)
**Firm_Age**		-0.054***		-0.046***
		(-18.09)		(-15.66)
**Capex**		-0.085***		-0.077***
		(-4.12)		(-3.77)
**Outside_Dir**		-0.010		-0.005
		(-0.45)		(-0.24)
**Board_Size**		-0.001		-0.001
		(-1.29)		(-1.45)
**Mas**		0.050***		0.038***
		(4.59)		(3.49)
**Constant**	0.060***	0.056	0.075***	0.096**
	(87.12)	(1.15)	(109.29)	(2.00)
**Year fixed effect**	Yes	Yes	Yes	Yes
**Firm fixed effect**	Yes	Yes	Yes	Yes
**N**	12,848	12,848	12,848	12,848
**adj. R2**	0.54	0.58	0.60	0.64

Note: This table presents changes in the accounting conservatism of pledging firms after the implementation of the Provisions. The dependent variable is *C_score* in columns 1 and 2 and *Cons* in columns 3 and 4. We regressed the panel data using the reghdfe command. We controlled for the firm and year fixed effects for unobserved time-invariant firm-specific characteristics. The t-statistics are in brackets. See the S1 Appendix for variable definitions.

***, **, and * indicate two-tailed significance at the 1%, 5%, and 10% levels, respectively.

Regarding the control variables, *Lev* is positively associated with *C_Score* and *Cons*, indicating that firms with greater financial leverage adopted more conservative accounting. The coefficient of *MB* is significantly negative, which suggests that firms with a lower market-to-book value also employed more conservative accounting. The above findings were consistent with prior studies [71, 80, 81].

### 4.2. Effect of the pledged share ratio

In this section, we examine the effect of the pledged share ratio on accounting conservatism. As shown in Table 4, columns 1 and 3, the coefficient of *Post×P_Ratio* is significantly positive, indicating that after the implementation of the Provisions, accounting conservatism was higher in firms with a higher average ratio of pledged shares; this result did not shift when changing the variable to *maxP_Ratio*, the regression results are similar to those in the previous analysis. Thus, the findings in Table 4 support Hypothesis 2. The results suggest that creditors had a higher power to trigger margin calls when the proportion of pledged shares was higher. Following the Provisions, pledge creditors were more likely to trigger margin calls if they were concerned about agency costs. Therefore, higher accounting conservatism seems to be a strategy for debtors with a higher level of pledged shares to mitigate agency problems.

**Table 4:** Effect of the pledged share ratio.

Variable	*C_score*		*Cons*	
	(1)	(2)	(3)	(4)
**P_Ratio×Post**	0.176***		0.226***	
	(5.14)		(6.66)	
**maxP_Ratio×Post**		0.092***		0.112***
		(5.93)		(7.32)
**Size**	0.004*	0.004*	0.002	0.002
	(1.91)	(1.67)	(1.09)	(0.78)
**Lev**	0.132***	0.131***	0.155***	0.154***
	(17.77)	(17.65)	(21.06)	(20.92)
**Growth**	-0.000	-0.000	-0.000	0.000
	(-0.15)	(-0.02)	(-0.03)	(0.12)
**CFO**	0.038***	0.038***	0.035***	0.036***
	(3.78)	(3.84)	(3.59)	(3.65)
**Div_Yield**	0.182***	0.190***	0.130*	0.139**
	(2.70)	(2.81)	(1.94)	(2.08)
**Big4**	0.003	0.003	0.004	0.004
	(0.46)	(0.49)	(0.61)	(0.66)
**MB**	-0.006***	-0.006***	-0.009***	-0.009***
	(-8.16)	(-8.07)	(-11.63)	(-11.52)
**Litigation**	0.008***	0.007***	0.007***	0.007***
	(3.74)	(3.59)	(3.60)	(3.42)
**SOE**	0.005	0.003	0.003	0.002
	(0.85)	(0.62)	(0.58)	(0.31)
**Firm_Age**	-0.053***	-0.054***	-0.045***	-0.046***
	(-17.87)	(-18.10)	(-15.33)	(-15.62)
**Capex**	-0.085***	-0.086***	-0.077***	-0.077***
	(-4.13)	(-4.16)	(-3.78)	(-3.79)
**Outside_Dir**	-0.011	-0.011	-0.006	-0.007
	(-0.48)	(-0.52)	(-0.28)	(-0.33)
**Board_Size**	-0.001	-0.001	-0.001	-0.001
	(-1.34)	(-1.26)	(-1.54)	(-1.43)
**Mas**	0.048***	0.050***	0.035***	0.037***
	(4.45)	(4.57)	(3.29)	(3.43)
**Constant**	0.042	0.055	0.078	0.094**
	(0.87)	(1.13)	(1.62)	(1.96)
**Year fixed effect**	Yes	Yes	Yes	Yes
**Firm fixed effect**	Yes	Yes	Yes	Yes
**N**	12,848	12,848	12,848	12,848
**adj. R2**	0.58	0.58	0.64	0.64

Note: This table shows how the ratio of pledged shares affected accounting conservatism after the implementation of the Provisions. We used two variables to measure the proportion of pledged shares: P_Ratio is the average ratio of pledged shares to the total shares for all pledges in a listed firm, and maxP_Ratio is the largest ratio of accumulated shares pledged by any shareholders to the total shares in a listed firm. The dependent variable is *C_score* in columns 1 and 2 and *Cons* in columns 3 and 4. We regressed the panel data using the reghdfe command. We controlled for the firm and year fixed effects for unobserved time-invariant firm-specific characteristics. The *t*-statistics are in brackets. See the S1 Appendix for variable definitions.

***, **, and * indicate two-tailed significance at the 1%, 5%, and 10% levels, respectively.

### 4.3. The effect of the remaining pledge period

In this section, we examine the effect of the remaining pledge period on accounting conservatism. As presented in Table 5, columns 1 and 3, the coefficient of *Post×P_Time* is significantly positive, indicating that accounting conservatism was higher (on average) in firms whose pledges had more years remaining than in other firms. This finding suggests that pledging shareholders were more likely to exert governance over firms with a longer (vs. shorter) remaining maturity period of pledged shares after the implementation of the Provisions. We obtained a similar result when changing the variable to the remaining pledge period of the firm’s longest pledge. The coefficient of *Post×longP_Time* is significantly positive in all columns, suggesting that the remaining pledge period of the longest pledge was positively associated with accounting conservatism in pledging firms after the implementation of the Provisions. These findings support Hypothesis 3. The results imply that creditors’ concerns about agency costs increased when the remaining maturity period of pledged shares was long after the implementation of the Provisions. Given that enhancing accounting conservatism is an effective strategy to alleviate creditors’ concern about agency costs [47, 48], firms with a longer maturity period of pledged shares are more likely to exhibit a higher level of accounting conservatism.

**Table 5:** The effect of the remaining pledge period.

Variable	*C_score*		*Cons*	
	(1)	(2)	(3)	(4)
**P_Time×Post**	0.005***		0.006***	
	(4.24)		(5.23)	
**longP_Time×Post**		0.004***		0.004***
		(4.92)		(5.54)
**Size**	0.003	0.003	0.001	0.001
	(1.52)	(1.43)	(0.60)	(0.52)
**Lev**	0.131***	0.130***	0.153***	0.153***
	(17.53)	(17.45)	(20.76)	(20.68)
**Growth**	-0.000	-0.000	-0.000	0.000
	(-0.16)	(-0.08)	(-0.05)	(0.03)
**CFO**	0.037***	0.037***	0.035***	0.034***
	(3.73)	(3.70)	(3.52)	(3.48)
**Div_Yield**	0.190***	0.195***	0.140**	0.144**
	(2.81)	(2.88)	(2.08)	(2.16)
**Big4**	0.003	0.003	0.004	0.004
	(0.52)	(0.51)	(0.69)	(0.68)
**MB**	-0.006***	-0.006***	-0.009***	-0.009***
	(-8.13)	(-8.08)	(-11.59)	(-11.54)
**Litigation**	0.008***	0.007***	0.007***	0.007***
	(3.72)	(3.67)	(3.57)	(3.52)
**SOE**	0.004	0.003	0.002	0.002
	(0.72)	(0.63)	(0.43)	(0.34)
**Firm_Age**	-0.054***	-0.054***	-0.046***	-0.046***
	(-18.12)	(-18.15)	(-15.65)	(-15.66)
**Capex**	-0.086***	-0.087***	-0.077***	-0.078***
	(-4.16)	(-4.20)	(-3.80)	(-3.83)
**Outside_Dir**	-0.009	-0.009	-0.005	-0.004
	(-0.43)	(-0.39)	(-0.22)	(-0.18)
**Board_Size**	-0.001	-0.001	-0.001	-0.001
	(-1.24)	(-1.21)	(-1.41)	(-1.38)
**Mas**	0.049***	0.049***	0.036***	0.037***
	(4.52)	(4.55)	(3.37)	(3.40)
**Constant**	0.062	0.066	0.103**	0.106**
	(1.27)	(1.36)	(2.14)	(2.20)
**Year fixed effect**	Yes	Yes	Yes	Yes
**Firm fixed effect**	Yes	Yes	Yes	Yes
**N**	12,848	12,848	12,848	12,848
**adj. R2**	0.58	0.58	0.64	0.64

Note: This table shows the effect of the remaining pledge period on accounting conservatism after the implementation of the Provisions. We used two variables to proxy the remaining pledge time: P_Time indicates the average number of remaining years of the pledges in a listed firm, and longP_Time is the largest number of remaining pledge years among large shareholders’ pledges for a treatment group firm in the year when the Provisions were executed. The dependent variable is *C_score* in columns 1 and 2 and *Cons* in columns 3 and 4. We regressed the panel data using the reghdfe command. We controlled for firm and year fixed effects for unobserved time-invariant firm-specific characteristics. The t-statistics are in brackets. See the S1 Appendix for variable definitions.

***, **, and * indicate two-tailed significance at the 1%, 5%, and 10% levels, respectively.

### 4.4. Effect of creditor concentration

In this section, we investigate the effect of creditor concentration on the change in pledging firms’ accounting conservatism after the implementation of the Provisions. In Table 6, the coefficients of *Post×CN* are significantly positive in all of the columns. The results suggest that the concentration of pledge creditors was significantly associated with pledging firms’ higher accounting conservatism post-implementation. These findings support Hypothesis 4. Following the enactment of the Provisions, concentrated pledge creditors had greater power (than their less concentrated peers) to enforce margin calls when they were concerned about agency problems.

**Table 6:** The effect of creditor concentration.

Variable	*C_score*		*Cons*	
	(1)	(2)	(3)	(4)
**CN×Post**	0.344***	0.339***	0.414***	0.396***
	(5.77)	(5.90)	(6.99)	(6.97)
**Size**		0.004*		0.002
		(1.70)		(0.81)
**Lev**		0.131***		0.154***
		(17.66)		(20.92)
**Growth**		-0.000		0.000
		(-0.02)		(0.10)
**CFO**		0.038***		0.036***
		(3.85)		(3.66)
**Div_Yield**		0.191***		0.140**
		(2.82)		(2.09)
**Big4**		0.003		0.004
		(0.46)		(0.63)
**MB**		-0.006***		-0.009***
		(-8.12)		(-11.58)
**Litigation**		0.007***		0.007***
		(3.60)		(3.43)
**SOE**		0.004		0.002
		(0.68)		(0.38)
**Firm_Age**		-0.053***		-0.045***
		(-18.00)		(-15.48)
**Capex**		-0.084***		-0.076***
		(-4.10)		(-3.72)
**Outside_Dir**		-0.012		-0.008
		(-0.54)		(-0.36)
**Board_Size**		-0.001		-0.001
		(-1.28)		(-1.46)
**Mas**		0.049***		0.036***
		(4.49)		(3.33)
**Constant**	0.060***	0.054	0.075***	0.093*
	(110.28)	(1.11)	(139.68)	(1.93)
**Year fixed effect**	Yes	Yes	Yes	Yes
**Firm fixed effect**	Yes	Yes	Yes	Yes
**N**	12,848	12,848	12,848	12,848
**adj. R2**	0.55	0.58	0.60	0.64

Note: This table presents the effect of creditor concentration on the change in pledging firms’ accounting conservatism after the implementation of the Provisions. The dependent variable is *C_score* in columns 1 and 2 and *Cons* in columns 3 and 4. We regressed the panel data using the reghdfe command. We controlled for the firm and year fixed effects for unobserved time-invariant firm-specific characteristics. The t-statistics are in brackets. See the S1 Appendix for variable definitions.

***, **, and * indicate two-tailed significance at the 1%, 5%, and 10% levels, respectively.

## 5. Additional analysis

### 5.1. Pledge creditors’ pressure on shareholders: Frequency of margin calls

A margin call occurs when there is a high risk that the value of pledged shares will not sufficiently offset the loan payment. When a margin call is triggered, pledge creditors require the pledge shareholders to deposit additional money or shares into the account to keep it at the maintenance margin. Before the Provisions, a margin call was triggered when stock prices fell below the liquidation line. Debtors’ failure to meet the required margin empowers creditors to sell their pledged shares and prevent default loss. After the implementation of the Provisions, pledge creditors are no longer able to immediately sell their pledged shares to close out the loan. Thus, creditors are more likely to trigger a margin call to stave off default if they become concerned about debtors’ risk-shifting actions. Therefore, we predict that after the implementation of share reduction provisions, the relationship between accounting conservatism and the frequency of margin calls is significant and negative.

We defined the dependent variable, *Margin_Call*_*t*_, as the number of times a margin call was triggered in the sample year. We performed a regression on accounting conservatism in the previous year, but only for share-pledging firms (as there were no margin calls for non-pledging firms). We also added another control variable to the regression, *Pledge_Rate*, which refers to the ratio of pledged shares to the total shares, on the basis that the proportion of pledged shares affects the frequency of margin calls. As shown in Table 7, almost all of the coefficients of *Post×C_Score*_*t-1*_ and *Post×Cons*_*t-1*_ are significant and negative. The empirical evidence is consistent with Hypothesis 1: After the Provisions were implemented, conservative financial reporting becomes an effective way to release low-risk signals to creditors and alleviate pledge creditors’ concerns about shareholder-creditor conflicts, thereby avoiding frequent margin calls.

**Table 7:** Frequency of margin calls.

Variable	*Margin_Call*			
	(1)	(2)	(3)	(4)
**Post**	7.013***	7.270***	5.370***	5.339***
	(5.24)	(4.19)	(6.81)	(4.03)
**C_Score**_***t*−1**_	19.070**	29.066***		
	(2.39)	(3.22)		
**Post×C_Score**_***t*−1**_	-16.845**	-29.165***		
	(-2.01)	(-3.08)		
**Cons**_***t*−1**_			3.640	15.073*
			(0.43)	(1.75)
**Pledge×Cons**_***t*−1**_			-3.877	-13.847*
			(-0.47)	(-1.68)
**Pledge_Rate**		-0.691***		-0.692***
		(-6.33)		(-6.34)
**Size**		-0.180		-0.203
		(-0.36)		(-0.41)
**Lev**		0.057		0.110
		(0.04)		(0.08)
**Growth**		0.020		0.033
		(0.08)		(0.13)
**CFO**		-0.119		-0.047
		(-0.06)		(-0.03)
**Div_Yield**		1.043		1.063
		(0.10)		(0.10)
**Big4**		-0.695		-0.670
		(-1.34)		(-1.29)
**MB**		-1.177***		-1.164***
		(-4.03)		(-4.06)
**Litigation**		-0.302		-0.308
		(-1.08)		(-1.10)
**SOE**		-0.233		-0.204
		(-0.63)		(-0.55)
**Firm_Age**		0.450		0.325
		(0.55)		(0.39)
**Capex**		-0.832		-0.731
		(-0.25)		(-0.22)
**Outside_Dir**		4.227		3.982
		(1.31)		(1.23)
**Board_Size**		0.212		0.207
		(1.56)		(1.53)
**Mas**		2.048		1.978
		(1.54)		(1.51)
**Year fixed effect**	Yes	Yes	Yes	Yes
**Firm fixed effect**	Yes	Yes	Yes	Yes
**N**	6,424	6,424	6,424	6,424
**adj. R2**	0.14	0.15	0.14	0.15

Note: This table presents the impact of accounting conservatism on the frequency of margin calls in pledging firms. The dependent variable is *Margin_Call*, which indicates the number of times a margin call is triggered in year t. The independent variables, *C_Score* and *Cons*, represent the accounting conservatism in year t–1. We used feologit regression as the main method. We controlled for the firm and year fixed effects. The t-statistics are in brackets. See the S1 Appendix for variable definitions.

***, **, and * indicate two-tailed significance at the 1%, 5%, and 10% levels, respectively.

### 5.2. Stock price crash risk

Pledging shareholders determine their optimal level of conservatism by weighing the tradeoff between benefits and costs. Conservative reporting is an effective strategy to mitigate creditors’ concerns about agency problems and prevent additional margin calls. However, the share price is also critical for share pledged loans. Prior to the Provisions, pledging shareholders had an incentive to boost the stock price. By preventing the price from falling below the liquidation line, they could avoid forced sales and the transfer of control rights. The Provisions imposed limits on share reduction, compelling shareholders to risk potential losses from stock price crashes because of the inability to sell shares promptly. If conservative reporting endangers the downward risk of the stock price, pledging shareholders may hesitate to improve accounting conservatism. We assume that the crash risk of the stock price decreased in pledging firms after the implementation of the Provisions due to firms’ more conservative reporting. First, accounting conservatism represents efficient corporate governance, which can mitigate the risk of stock price crashes [17, 82]. Second, the risk of a crash increases when managers conceal negative information, as the sudden disclosure of this information substantially lowers stock prices [83]. Accounting conservatism limits managers’ motivation and ability to exaggerate performance or hide unfavorable news, which, in turn, reduces stock price crash risk [14, 53, 54].

Following Xu et al.(2021), we used the variable *DUVOL* to proxy the probability of a stock price crash [84]; this variable is the natural logarithm of the ratio of the standard deviation on the down weeks to the standard deviation on the up weeks. Down (up) weeks are defined as the weeks with firm-specific weekly returns below (above) the annual mean. The larger the *DUVOL* value, the greater the stock price crash risk. Table 8 reports the empirical results for the stock price crash risk. In columns 1 and 2, the coefficients of *Pledge×Post×C_Score* are significant and negative at the 1% level, indicating that, after the implementation of the Provisions, the higher accounting conservatism in pledging firms led to a lower stock price crash risk. The results in columns 3 and 4 corroborate this result, with the coefficients of *Pledge×Post×Cons* being significantly negative at the 5% level and 10% level, respectively. The above findings suggest that after the implementation of the Provisions, pledging shareholders increased accounting conservatism without raising the risk of a stock price collapse.

**Table 8:** Stock price crash risk.

Variable	*DUVOL*_*t*+1_			
	(1)	(2)	(3)	(4)
**Pledge×Post**	0.058*	0.061**	0.044	0.043
	(1.87)	(1.98)	(1.41)	(1.37)
**Pledge×C_Score**	2.298***	2.220***		
	(2.67)	(2.59)		
**Post×C_Score**	2.493***	1.061		
	(3.48)	(1.41)		
**Pledge×Post×C_Score**	-2.414***	-2.365***		
	(-2.78)	(-2.73)		
**C_Score**	-2.751***	-1.243*		
	(-3.87)	(-1.66)		
**Pledge×Cons**			1.000*	0.823
			(1.91)	(1.57)
**Post×Cons**			1.274***	0.412
			(3.07)	(0.96)
**Pledge×Post×Cons**			-1.028**	-0.887*
			(-1.96)	(-1.70)
**Cons**			-1.611***	-0.626
			(-3.82)	(-1.43)
**Size**		0.108***		0.106***
		(5.56)		(5.48)
**Lev**		-0.126*		-0.127*
		(-1.79)		(-1.85)
**Growth**		-0.002		-0.002
		(-0.29)		(-0.26)
**CFO**		-0.050		-0.051
		(-0.55)		(-0.57)
**Div_Yield**		-3.247***		-3.257***
		(-5.31)		(-5.34)
**Big4**		-0.065		-0.066
		(-1.16)		(-1.18)
**MB**		0.067***		0.066***
		(9.03)		(8.98)
**Litigation**		0.008		0.008
		(0.46)		(0.43)
**SOE**		0.018		0.017
		(0.38)		(0.34)
**Firm_Age**		-0.007		-0.006
		(-0.24)		(-0.24)
**Capex**		0.166		0.168
		(0.89)		(0.90)
**Outside_Dir**		0.171		0.175
		(0.86)		(0.88)
**Board_Size**		0.005		0.005
		(0.70)		(0.69)
**Mas**		0.175*		0.172*
		(1.78)		(1.75)
**Constant**	0.060***	0.054	0.075***	0.093*
	(110.28)	(1.11)	(139.68)	(1.93)
**Year fixed effect**	Yes	Yes	Yes	Yes
**Firm fixed effect**	Yes	Yes	Yes	Yes
**N**	12,848	12,848	12,848	12,848
**adj. R2**	0.14	0.15	0.14	0.15

Note: This table presents the stock price crash risk of pledging firms after the implementation of the Provisions. The dependent variable is *DUVOL*_*t*+1_. We regressed the panel data using the reghdfe command and controlled for firm and year fixed effects. The t-statistics are in brackets. See the S1 Appendix for variable definitions.

***, **, and * indicate two-tailed significance at the 1%, 5%, and 10% levels, respectively.

### 5.3. Effect of controlling shareholders as pledgers

The governance effect of pledging on accounting conservatism depends on the characteristics of the pledgers. Since the Provisions restricted pledge creditors’ cashing-out behavior, we examined the impact of share pledging on corporate financial reporting when the major shareholders had pledged shares on the enforcement date of the Provisions. Controlling shareholders who have pledged shares are more inclined to increase accounting conservatism after the implementation of the Provisions. First, controlling shareholders temporarily lose their cash flow rights when they pledge shares, but they retain their control rights. The control right enables controlling shareholders to modify the firm’s accounting policies [7, 30]. Second, controlling shareholders have a significant motivation to utilize corporate resources to prevent a margin call, since it could undermine shareholders’ voting and control rights [5, 33]. We divided the treatment group into two samples: one in which controlling shareholders are the pledgers and one in which they are not. In columns 1 and 2 of Table 9, the coefficients of *Pledge×Post* are significant and positive at the 1% level, but they are not significant in columns 3 and 4. In other words, our main findings were only significant when the controlling shareholders were the pledgers. This suggests that controlling pledgers are more likely than other shareholders to increase accounting conservatism in order to avoid losing control over the firm.

**Table 9:** The effect of controlling shareholders as pledgers.

Variable	Controlling shareholders as pledgers		Non-controlling shareholders as pledgers	
	*C_score*	*Cons*	*C_score*	*Cons*
	(1)	(2)	(3)	(4)
**Pledge×Post**	0.011***	0.013***	0.001	0.004
	(4.89)	(5.88)	(0.37)	(1.28)
**Size**	0.004*	0.003	0.007**	0.005
	(1.74)	(1.19)	(2.13)	(1.55)
**Lev**	0.123***	0.144***	0.144***	0.163***
	(16.77)	(19.90)	(13.48)	(15.45)
**Growth**	-0.000	-0.000	-0.001	-0.001
	(-0.59)	(-0.41)	(-0.85)	(-0.74)
**CFO**	0.040***	0.040***	0.019	0.020
	(3.99)	(4.08)	(1.43)	(1.52)
**Div_Yield**	0.188***	0.142**	0.264**	0.196
	(2.64)	(2.01)	(2.16)	(1.62)
**Big4**	0.003	0.002	-0.005	-0.006
	(0.37)	(0.27)	(-0.48)	(-0.65)
**MB**	-0.007***	-0.009***	-0.005***	-0.007***
	(-8.79)	(-11.56)	(-4.33)	(-6.64)
**Litigation**	0.006***	0.006***	0.013***	0.013***
	(3.05)	(2.73)	(4.23)	(4.08)
**SOE**	-0.030***	-0.033***	0.014**	0.013**
	(-3.58)	(-4.02)	(2.55)	(2.39)
**Firm_Age**	-0.050***	-0.042***	-0.045***	-0.036***
	(-16.94)	(-14.44)	(-10.11)	(-8.27)
**Capex**	-0.032	-0.027	-0.078***	-0.082***
	(-1.60)	(-1.36)	(-2.75)	(-2.93)
**Outside_Dir**	-0.025	-0.019	0.004	-0.002
	(-1.10)	(-0.86)	(0.12)	(-0.06)
**Board_Size**	-0.002*	-0.002**	-0.001	-0.001
	(-1.91)	(-1.98)	(-0.67)	(-0.67)
**Mas**	0.040***	0.029***	0.041***	0.036**
	(3.79)	(2.76)	(2.84)	(2.53)
**Constant**	0.062	0.084*	-0.059	-0.019
	(1.29)	(1.75)	(-0.82)	(-0.27)
**Year fixed effect**	Yes	Yes	Yes	Yes
**Firm fixed effect**	Yes	Yes	Yes	Yes
**N**	11,189	11,189	5,130	5,130
**adj. R2**	0.56	0.62	0.57	0.64

Note: This table shows the regression results when pledgers are controlling shareholders (columns 1 and 2) and non-controlling shareholders (columns 3 and 4). We regressed the panel data using the reghdfe command. We controlled for firm and year fixed effects for unobserved time-invariant firm-specific characteristics. The t-statistics are in brackets. See the S1 Appendix for variable definitions.

***, **, and * indicate two-tailed significance at the 1%, 5%, and 10% levels, respectively.

### 5.4. Effect of types of pledge creditor

In this section, we investigate the effect of various pledge creditors on pledging firms’ accounting conservatism following the Provisions’ implementation. In our sample, the pledge creditors mainly consisted of banks, security companies, and trust companies. To clearly identify the effect of each type of pledge creditor, we divided the treatment group into three samples: One for all bank-related observations; the second for security companies, and the third for trust companies. In general, lenders require a timely disclosure of bad news so they can take preventive action [85]; however, managers tend to withhold bad news [86]. Thus, pledge creditors set tight pledge covenants to constrain certain firm policies and scrutinize firms’ performance closely, thus protecting the lenders’ wealth from being transferred to shareholders [71]. Banks have more bargaining power than other types of creditors [87] and thus more power to trigger margin calls. In Table 10, columns 1 and 2 present the results for banks, columns 3 and 4 show the results for security companies, and columns 5 and 6 present the results for trust companies. The coefficients of *Pledge×Post* are significant and positive at the 5% level in columns 1 and 2 but are not significant in the remaining columns. In other words, our main findings are only significant for the sample in which banks are the pledge creditors. This suggests that banks are more powerful and capable of compelling pledging shareholders to enhance their accounting conservatism.

**Table 10:** The effects of different pledge creditors.

Variable	*Bank*		*Security Company*		*Trust Company*	
	*C_score*	*Cons*	*C_score*	*Cons*	*C_score*	*Cons*
	(1)	(2)	(3)	(4)	(5)	(6)
**Pledge×Post**	0.009**	0.010**	-0.002	0.003	0.013	0.017
	(2.09)	(2.54)	(-0.76)	(1.05)	(1.12)	(1.43)
**Size**	0.006*	0.005	0.006**	0.004	0.004	0.003
	(1.72)	(1.47)	(2.09)	(1.58)	(1.11)	(0.93)
**Lev**	0.129***	0.147***	0.132***	0.153***	0.126***	0.145***
	(11.81)	(13.63)	(14.29)	(16.74)	(10.88)	(12.63)
**Growth**	0.000	0.000	0.000	0.000	0.000	0.000
	(0.22)	(0.45)	(0.39)	(0.60)	(0.17)	(0.51)
**CFO**	0.023*	0.025*	0.032***	0.032***	0.019	0.022
	(1.70)	(1.90)	(2.71)	(2.72)	(1.35)	(1.56)
**Div_Yield**	0.293***	0.225**	0.233***	0.185**	0.292***	0.222**
	(3.24)	(2.52)	(2.81)	(2.25)	(2.99)	(2.30)
**Big4**	0.008	0.008	0.003	0.003	0.006	0.006
	(1.11)	(1.18)	(0.38)	(0.49)	(0.77)	(0.81)
**MB**	-0.006***	-0.009***	-0.008***	-0.011***	-0.007***	-0.010***
	(-5.89)	(-8.64)	(-8.80)	(-11.69)	(-6.24)	(-8.87)
**Litigation**	0.003	0.003	0.007***	0.006**	0.003	0.003
	(1.17)	(0.95)	(2.63)	(2.48)	(1.05)	(0.87)
**SOE**	-0.017**	-0.020**	-0.004	-0.007	-0.017*	-0.020**
	(-1.98)	(-2.33)	(-0.56)	(-0.93)	(-1.77)	(-2.08)
**Firm_Age**	-0.055***	-0.047***	-0.050***	-0.042***	-0.054***	-0.045***
	(-14.13)	(-12.03)	(-14.62)	(-12.43)	(-13.22)	(-11.11)
**Capex**	-0.094***	-0.083***	-0.079***	-0.073***	-0.092***	-0.082***
	(-3.45)	(-3.09)	(-3.24)	(-3.02)	(-3.21)	(-2.87)
**Outside_Dir**	-0.060**	-0.064**	-0.030	-0.032	-0.059**	-0.064**
	(-2.12)	(-2.28)	(-1.16)	(-1.22)	(-2.01)	(-2.21)
**Board_Size**	-0.004***	-0.004***	-0.002*	-0.002**	-0.003***	-0.004***
	(-3.17)	(-3.54)	(-1.91)	(-2.26)	(-3.02)	(-3.40)
**Mas**	0.098***	0.081***	0.060***	0.047***	0.108***	0.091***
	(5.56)	(4.65)	(4.74)	(3.78)	(5.94)	(5.06)
**Constant**	0.066	0.087	0.015	0.050	0.101	0.118
	(0.91)	(1.22)	(0.24)	(0.79)	(1.30)	(1.55)
**Year fixed effect**	Yes	Yes	Yes	Yes	Yes	Yes
**Firm fixed effect**	Yes	Yes	Yes	Yes	Yes	Yes
**N**	7,146	7,146	8,898	8,898	6,416	6,416
**adj. R2**	0.58	0.63	0.57	0.63	0.57	0.63

Note: This table shows the regression results when the pledge creditors are banks, security companies, and trust companies. Columns 1 and 2 present the results for banks, columns 3 and 4 show the results for security companies, and columns 5 and 6 present the results for trust companies. We regressed the panel data using the reghdfe command. We controlled for firm and year fixed effects for unobserved time-invariant firm-specific characteristics. The t-statistics are in brackets. See the S1 Appendix for variable definitions.

***, **, and * indicate two-tailed significance at the 1%, 5%, and 10% levels, respectively.

## 6. Robustness tests

### 6.1. Alternative definition of the control group

Above, we defined the control group as firms in which major shareholders did not pledge shares on the Provisions’ exact implementation date; therefore, this group may include major shareholders who pledged shares before or after the implementation date, which may yield inaccurate empirical results. Thus, we constructed two additional control groups to perform a robustness test. The first group consists of the cases in the original control group in which major shareholders had pledged shares and repaid the debt before the implementation date and did not pledge shares after the implementation (CG_1). The second group consists of the cases in the original control group in which major shareholders did not pledge shares during the sample period (CG_2). The treatment group is the same as before. The results in Table 11 show that the coefficients of *Pledge×Post* are significantly positive in all of the columns, suggesting that our main findings were not influenced by these alternative definitions of the control group.

**Table 11:** Alternative definitions of the control group.

Variable	*C_score*		*Cons*	
	CG_1	CG_2	CG_1	CG_2
	(1)	(2)	(3)	(4)
**Pledge×Post**	0.028***	0.009***	0.023***	0.011***
	(4.43)	(3.53)	(3.75)	(4.25)
**Size**	0.007	0.005*	0.007	0.003
	(1.28)	(1.78)	(1.44)	(1.00)
**Lev**	0.072***	0.117***	0.094***	0.140***
	(3.70)	(12.08)	(4.88)	(14.60)
**Growth**	-0.001	0.000	-0.001	0.000
	(-0.58)	(0.38)	(-0.59)	(0.49)
**CFO**	-0.001	0.039***	0.002	0.036***
	(-0.02)	(3.13)	(0.07)	(2.97)
**Div_Yield**	0.250	0.275***	0.181	0.221***
	(1.38)	(3.34)	(1.02)	(2.73)
**Big4**	-0.017	-0.003	-0.017	0.000
	(-0.72)	(-0.45)	(-0.74)	(0.02)
**MB**	-0.004*	-0.004***	-0.006***	-0.007***
	(-1.80)	(-3.97)	(-2.99)	(-7.01)
**Litigation**	0.005	0.008***	0.003	0.008***
	(0.93)	(3.13)	(0.62)	(3.09)
**SOE**	0.021	0.003	0.013	0.001
	(1.27)	(0.46)	(0.83)	(0.16)
**Firm_Age**	-0.119***	-0.068***	-0.123***	-0.060***
	(-8.20)	(-14.68)	(-8.67)	(-13.21)
**Capex**	-0.141**	-0.104***	-0.119*	-0.100***
	(-2.08)	(-3.75)	(-1.77)	(-3.67)
**Outside_Dir**	-0.050	-0.016	-0.021	-0.014
	(-0.87)	(-0.60)	(-0.37)	(-0.52)
**Board_Size**	-0.004	-0.001	-0.004*	-0.001
	(-1.65)	(-1.00)	(-1.67)	(-1.01)
**Mas**	-0.021	0.044**	-0.026	0.027
	(-0.49)	(2.47)	(-0.63)	(1.55)
**Constant**	0.233*	0.081	0.234*	0.123**
	(1.91)	(1.33)	(1.95)	(2.05)
**Year fixed effect**	Yes	Yes	Yes	Yes
**Firm fixed effect**	Yes	Yes	Yes	Yes
**N**	1,498	7,778	1,498	7,778
**adj. R2**	0.62	0.60	0.67	0.65

Note: This table presents the regression results with adjusted control groups. In the first adjusted group, we retained the cases in the original control group in which large shareholders pledged shares and repaid the debt before the Provisions’ implementation and did not pledge afterward (CG_1); columns 1 and 3 capture that regression. In the second adjusted group, we retained the sample in the original control group in which large shareholders did not pledge shares during the sample period (CG_2); columns 2 and 4 depict that regression. The dependent variable is *C_score* in columns 1 and 2 and *Cons* in columns 3 and 4. We regressed the panel data using the reghdfe command. We controlled for firm and year fixed effects for unobserved time-invariant firm-specific characteristics. The t-statistics are in brackets. See the S1 Appendix for variable definitions.

***, **, and * indicate two-tailed significance at the 1%, 5%, and 10% levels, respectively.

### 6.2. Placebo test

**Fig 4:**
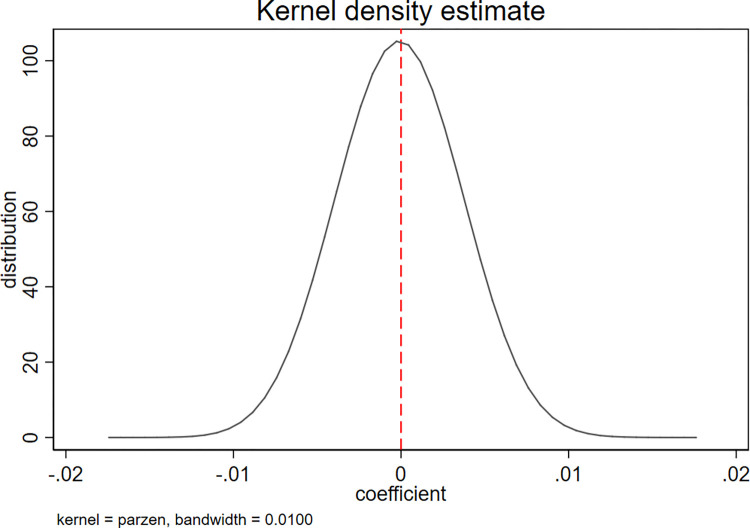
Placebo test for *C_score*.

**Fig 5:**
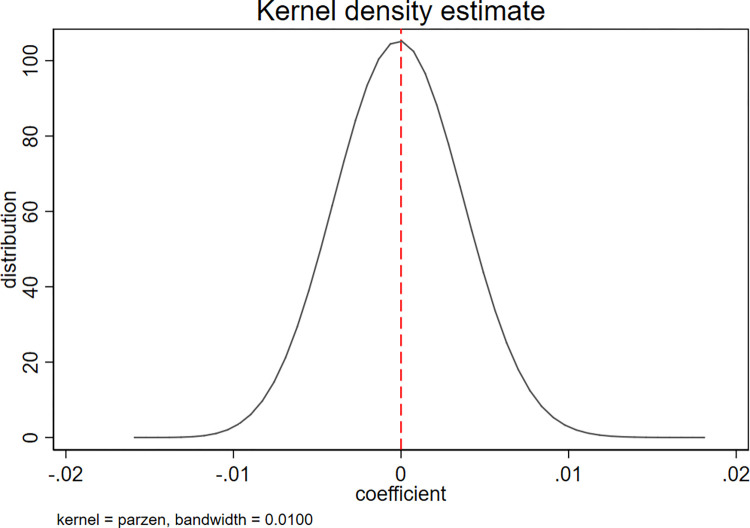
Placebo test for *Cons*.

Following prior studies [88, 89], we applied kernel density estimation in a placebo test of our DID analysis, aiming to exclude the impact of other potential events or factors on our empirical results. Figs 4 and 5 present the results of the placebo tests for the model specification. The placebo tests were based on a randomized sample from 500 simulations. For each simulation, we drew a random sample from the pool of all firms as the treatment group, then treated the remaining sample as the control group. The dependent variables are *C_Score* and *Cons*. The distribution of the coefficient of *Pledge×Post* is approximately normal, with a mean of 0, as shown in both figures. Our placebo tests did not generate a significant effect of the Provisions on the accounting conservatism of pledging firms; thus, we can confirm that our empirical results were due to the enactment of the Provisions rather than to other events or factors.

### 6.3. Excluding samples in which either executives or shareholders sell shares

Since the implementation of the Provisions, shareholders cannot immediately short their shares in large volumes. When shareholders and senior executives are about to undergo a share reduction, they have an incentive to increase accounting conservatism because it reduces the likelihood of downside risk in the stock price and increases the likelihood that the stock price will remain stable, meaning that they can sell the shares at a relatively high price. This may confound our prediction that pledging shareholders increase accounting conservatism to avoid margin call from creditors. To address this concern, we excluded observations from the treatment group in which any shareholders or senior executives shorted shares in the sample year. The results in Table 12 show that there are no significant changes in the coefficients of *Pledge×Post*, suggesting that our conclusions were not affected by senior executives and shareholders’ share reduction motivation.

**Table 12:** Exclusion of sample firms in which either executives or shareholders sell shares.

Variable	*C_score*		*Cons*	
	Senior Executives	Shareholders	Senior Executives	Shareholders
	(1)	(2)	(3)	(4)
**Pledge×Post**	0.016***	0.016***	0.019***	0.018***
	(7.27)	(5.40)	(8.42)	(6.16)
**Size**	0.007***	0.010***	0.006**	0.009***
	(3.16)	(3.35)	(2.57)	(3.04)
**Lev**	0.124***	0.106***	0.145***	0.125***
	(16.09)	(10.15)	(19.17)	(12.05)
**Growth**	-0.000	-0.000	-0.000	0.000
	(-0.58)	(-0.38)	(-0.54)	(0.17)
**CFO**	0.028***	0.015	0.026***	0.016
	(2.86)	(1.20)	(2.78)	(1.32)
**Div_Yield**	0.278***	0.236**	0.205***	0.155
	(3.69)	(2.39)	(2.76)	(1.58)
**Big4**	0.003	0.006	0.003	0.005
	(0.41)	(0.62)	(0.45)	(0.49)
**MB**	-0.004***	0.000	-0.007***	-0.003**
	(-4.99)	(0.11)	(-8.12)	(-2.29)
**Litigation**	0.008***	0.007**	0.008***	0.007**
	(3.72)	(2.42)	(3.54)	(2.30)
**SOE**	-0.012**	0.003	-0.013**	0.000
	(-1.98)	(0.39)	(-2.30)	(0.01)
**Firm_Age**	-0.052***	-0.045***	-0.044***	-0.039***
	(-16.59)	(-9.37)	(-14.18)	(-8.26)
**Capex**	-0.042**	-0.001	-0.042**	-0.008
	(-2.03)	(-0.04)	(-2.07)	(-0.30)
**Outside_Dir**	-0.025	-0.029	-0.020	-0.027
	(-1.03)	(-0.95)	(-0.86)	(-0.88)
**Board_Size**	-0.001	-0.001	-0.001	-0.001
	(-0.98)	(-0.78)	(-1.16)	(-1.03)
**Mas**	0.047***	0.067***	0.036***	0.054***
	(3.75)	(3.56)	(2.91)	(2.91)
**Constant**	-0.020	-0.103	0.006	-0.076
	(-0.40)	(-1.51)	(0.12)	(-1.13)
**Year fixed effect**	Yes	Yes	Yes	Yes
**Firm fixed effect**	Yes	Yes	Yes	Yes
**N**	10,341	5,996	10,341	5,996
**adj. R2**	0.59	0.64	0.65	0.68

Note: This table shows the results of the regression when we delete the sample firms from the treatment group in which any shareholders or senior executives sold shares in the sample year. We regressed the panel data using the reghdfe command while controlling for firm and year fixed effects. The t-statistics are in brackets. See the S1 Appendix for the variable definitions.

***, **, and * indicate two-tailed significance at the 1%, 5%, and 10% levels, respectively.

### 6.4. Other measures of accounting conservatism

We used three other methods to measure firms’ accounting conservatism. Following Ball and Shivakumar (2005), we estimated a piecewise-linear relation between cash flows and accruals and used the variable *ACF* to represent the extent of accounting conservatism [53]. Following Beaver and Ryan (2005), we calculated accounting conservatism as the understatement of the book value of net assets relative to their market value [90]. BMT is the additive inverse of the indicator, which represents a firm’s accounting conservatism. Following Tan (2013), we also used goodwill impairment to manifest conservatism. Goodwill impairment is a concrete example of heightened conservatism as it ensures that assets are not overstated and that potential losses are promptly recognized [15]. The results in Table 13 highlight the persistent significant changes in the coefficients of *Pledge×Post*, suggesting that our conclusion was robust to other measures of accounting conservatism.

**Table 13:** Other measures of accounting conservatism.

Variable	*ACF*		BTM		Goodwill	
	(1)	(2)	(3)	(4)	(5)	(6)
Pledge×Post	3.976***	4.227***	0.955***	0.643***	0.033***	0.026***
	(2.92)	(3.07)	(6.23)	(4.24)	(6.55)	(5.26)
Size		-1.719		0.205		-0.033***
		(-1.21)		(1.31)		(-5.73)
Lev		2.612		2.458***		0.066***
		(0.53)		(4.40)		(3.58)
Growth		0.273		-0.053		-0.010***
		(0.62)		(-1.17)		(-5.55)
CFO		-19.786***		0.445		0.045*
		(-3.03)		(0.65)		(1.75)
Div_Yield		-81.193*		-6.697		-0.219
		(-1.83)		(-1.41)		(-1.38)
Big4		-2.339		-0.051		0.018
		(-0.56)		(-0.12)		(1.14)
MB		1.231**		-0.220***		0.001
		(2.41)		(-3.48)		(0.61)
Litigation		0.082		0.346**		-0.001
		(0.06)		(2.40)		(-0.18)
SOE		-6.787*		-0.527		0.011
		(-1.90)		(-1.33)		(0.91)
Firm_Age		3.866**		12.143***		0.060***
		(1.98)		(12.76)		(7.49)
Capex		13.001		-0.562		0.140**
		(0.96)		(-0.35)		(2.58)
Outside_Dir		-2.298		0.600		-0.020
		(-0.16)		(0.39)		(-0.37)
Board_Size		0.470		0.056		0.002
		(0.82)		(0.94)		(1.08)
Mas		17.180**		-5.954***		-0.070**
		(2.40)		(-3.35)		(-2.43)
Constant	-2.904***	23.105	21.525***	-16.230***	-0.031***	0.537***
	(-6.66)	(0.72)	(419.82)	(-3.74)	(-17.65)	(4.16)
Year fixed effect	Yes	Yes	Yes	Yes	Yes	Yes
Firm fixed effect	Yes	Yes	Yes	Yes	Yes	Yes
N	12,698	12,698	8,054	8,054	7612	7612
adj. R2	0.19	0.19	0.97	0.97	0.09	0.11

Note: This table presents the changes in pledging firms’ accounting conservatism after the implementation of the Provisions, using three other definitions of “accounting conservatism”. The dependent variable is *ACF* in columns 1 and 2, *BTM* in columns 3 and 4 and *Goodwill* in columns 5 and 6. We regressed the panel data using the reghdfe command. We controlled for firm and year fixed effects for unobserved time-invariant firm-specific characteristics. The t-statistics are in brackets. See the S1 Appendix for variable definitions.

***, **, and * indicate two-tailed significance at the 1%, 5%, and 10% levels, respectively.

### 6.5. Instrumental variable

Despite our application of the PSM-DID method, the above results could still suffer from endogeneity concerns. Therefore, we constructed an instrumental variable and used the two-stage least squares method to further alleviate the endogeneity issue. The instrumental variable (*Trust*) was proxied by data on social trust (measured on a scale from 0 to 5) at the province level, taken from a survey conducted by the China Household Finance Survey and Research Center. We chose this instrumental variable for two reasons: First, social trust is strongly and positively associated with ease of access to institutional credit [91], and pledge requirements may be lower in areas with higher social trust. Second, social trust is not directly related to the accounting conservatism of a specific firm. In other words, *Trust* is correlated with the independent variable, but exogenous to the dependent variable. Following Cai et al. (2016), in the first stage, we estimated *Pledge×Post* by *Trust×Post*; in the second stage, we used the predicted *Pledge×Post* in the regression [89]. As shown in Table 14, we found that *Trust×Post* was negatively associated with *Pledge×Post* in the first stage, while the coefficients of the predicted *Pledge×Post* remained significant and positive in the second stage. Thus, our findings remained robust when using the instrumental variable method to alleviate the endogeneity problem.

**Table 14:** Instrumental variable.

Variable	First stage	Second stage	Second stage
	*Pledge*×*Post*	*C_score*	*Cons*
	(1)	(2)	(3)
**Pledge×Post**		0.173***	0.182***
		(3.721)	(3.878)
**Trust×Post**	-0.111***		
	(-5.70)		
**Size**	0.031***	-0.002	-0.004
	(2.97)	(-0.54)	(-1.22)
**Lev**	0.150***	0.106***	0.127***
	(4.17)	(8.94)	(10.65)
**Growth**	-0.011***	0.002*	0.002**
	(-3.52)	(1.83)	(1.98)
**CFO**	-0.069	0.048***	0.046***
	(-1.44)	(3.68)	(3.51)
**Div_Yield**	-0.459	0.269***	0.221**
	(-1.40)	(2.99)	(2.45)
**Big4**	-0.014	0.005	0.007
	(-0.45)	(0.67)	(0.79)
**MB**	-0.016***	-0.004***	-0.006***
	(-4.26)	(-3.04)	(-5.01)
**Litigation**	0.013	0.005*	0.005*
	(1.35)	(1.94)	(1.75)
**SOE**	0.159***	-0.022**	-0.025**
	(6.09)	(-2.19)	(-2.45)
**Firm_Age**	0.106***	-0.071***	-0.063***
	(7.34)	(-11.72)	(-10.45)
**Capex**	0.385***	-0.149***	-0.143***
	(3.86)	(-4.66)	(-4.45)
**Outside_Dir**	-0.099	0.005	0.0105
	(-0.93)	(0.19)	(0.37)
**Board_Size**	-0.006	-0.000	-0.000
	(-1.46)	(-0.07)	(-0.15)
**Mas**	-0.256***	0.092***	0.081***
	(-4.86)	(5.04)	(4.41)
**Year fixed effect**	Yes	Yes	Yes
**Firm fixed effect**	Yes	Yes	Yes
**Cragg–Donald F**	32.545		
**N**	12,848	12,848	12,848
**R2**	0.38	0.279	0.327
**F**	551.79	379.9	439.2

Note: This table presents the regression results when using an instrumental variable to alleviate the endogeneity issue. The instrumental variable (*Trust*) is proxied by data on social trust (measured on a scale from 0 to 5) at the province level, taken from a survey conducted by the China Household Finance Survey and Research Center. Column 1 presents the results of the first stage. Columns 2 and 3 show the results of the second stage, with the dependent variables of *C_score* and *Cons*, respectively. The *t*-statistics are in brackets. See the S1 Appendix for variable definitions.

***, **, and * indicate two-tailed significance at the 1%, 5%, and 10% levels, respectively.

## 7. Conclusions

This paper used changes to China’s stock-selling rules, enacted in 2017, as a natural experiment to uncover how pledge behavior affects firms’ accounting conservatism. The promulgation of the Provisions increased pledge creditors’ potential losses related to loan default. Thus, pledge creditors had an incentive to take proactive actions when they were worried about a firm’s agency costs. Under these circumstances, pledging shareholders increased accounting conservatism to alleviate creditors’ concerns and avoid additional margin calls.

First, we found that accounting conservatism increased in pledging firms after the implementation of the Provisions, which supports our predictions. Pledging shareholders pursued greater accounting conservatism to signal their risk-avoidance and alleviate creditors’ concerns about agency costs. This effect was pronounced in firms with a higher pledged share ratio, a longer period to maturity of the pledged shares, and more concentrated pledge creditors. Second, we found that higher accounting conservatism in pledging firms was associated with fewer margin calls. This finding supports our argument that conservative financial reporting is an effective way to alleviate pledge creditors’ concerns about shareholder-creditor conflicts and stop them from taking proactive actions. Third, we found that the Provisions decreased the risk of a stock price crash due to more conservative financial reporting. Fourth, our results show that the effect of governance on accounting conservatism was stronger when the controlling shareholders were the pledgers due to their authority to alter accounting policies and their motivation to retain control of the firm. Fifth, and relatedly, the effect of governance on accounting conservatism was stronger when the pledge creditors were banks, since they hold more bargaining power than other creditors. Importantly, our results remained stable when subjected to several robustness tests.

This paper extends the literature on financial reporting quality by highlighting that share pledging can have a positive influence on accounting conservatism so long as there are strict regulations that increase creditor’s concerns about agency costs. Furthermore, by utilizing a natural experiment enabled by the Provisions, we addressed the endogeneity problem while elucidating the impact of pledge creditors on the behavior of pledging shareholders. Finally, the results underline the importance of regulation. Share pledging has become prevalent worldwide [33]. However, corporate scandals linked to the activity have cast a shadow over corporate governance and attracted scrutiny from regulators [3, 4, 6, 7, 32]. The regulatory interventions in China’s pledge market offer a framework for how regulations incentivize the disclosure of reliable accounting information among pledging firms. These insights represent a roadmap for regulators worldwide who want to enhance regulation of the pledge market and prompt its healthy development. It is worth noting that China’s creditor regulations are not drastically different from other developed countries. In terms of rights, creditors in China, like they do elsewhere, assign a loan-to-value ratio and determine the number of shares pledged in the contract terms [58]. Contract terms may vary in different countries, but borrowers must maintain the loan-to-value ratio or they will face a margin call from the creditors. Creditors have a legal right to liquidate the pledged shares at market prices if the margin requirement is not promptly met. Our paper demonstrates that specific provisions increase creditors’ concerns about agency problems—and the resulting threat from creditors incentivizes pledgers to improve corporate governance and reduce debt cost. In sum, this analysis offers valuable insights into global financial systems and will hopefully prompt stakeholders to adopt effective governance measures to mitigate adverse outcomes.

## Supporting information

S1 Appendix Variable definitions. (DOCX)
