# Supplementary material for: Share pledge and accounting conservatism in share-pledging firms: Evidence from a natural experiment in China
Source: PLoS One. 2024 Jul 9;19(7):e0306899. doi: 10.1371/journal.pone.0306899 (PMC11233009; doi:10.1371/journal.pone.0306899)
Supplement: S1 Appendix — (DOCX) [file pone.0306899.s001.docx]

# **Appendix A. Variable definitions**

| **Variable** | **Definition** | **Data source** |
| --- | --- | --- |
| ***Dependent variables*** |  |  |
| **C_Score** | Increase in the timeliness of the recognition of bad news in accounting earnings relative to the recognition of good news. Calculated by using the measure developed by Khan and Watts (2009) [68] and Wu et al. (2022) [24]. A high value means more conservative. | CSMAR |
| **Cons** | Timeliness of the recognition of bad news in accounting earnings. Calculated by using the measure developed by García Lara et al. (2016). A high value means more conservative. | CSMAR |
| **DUVOL** | Natural logarithm of the ratio of the standard deviation on the down weeks to the standard deviation on the up weeks. Down (up) weeks are defined as the weeks with firm-specific weekly returns below (above) the annual mean. Calculated by using the measure developed by Xu et al. (2021) [84]. | NetEast Finance |
| **Margin_Call** | Number of times a margin call is triggered in the next year. | Choice |
| ***Independent variables*** |  |  |
| **Pledge** | Equals 1 when any large shareholder pledged more than 5% of the total outstanding shares as of the enforcement date of the Provisions, and 0 otherwise. | Choice |
| **Post** | Equals 1 if the observation is in 2018 or later, and 0 otherwise. | Calculated by author |
| **P_Ratio** | Average ratio of pledged shares to the total shares in a treatment group firm when the Provisions that restrict pledge creditors’ selling-out behavior are enforced. Obtained by calculating the mean of the pledge ratio. | Choice |
| **maxP_Ratio** | Largest ratio of accumulated shares pledged by any shareholder to the total shares in a treatment group firm in the year when the regulation is implemented.  $maxP\_Ratio={max}_{i=1}^{n}(\frac{{Accumulated shares pledged by any shareholder}_{i}}{{Total shares}_{i}})$ | Choice |
| **P_Time** | Average number of remaining years of all the pledges in a treatment group firm when the Provisions are enforced. Obtained by calculating the mean of the remaining years of all the pledges. | Choice |
| **longP_Time** | Largest number of remaining pledging years among all the pledges for a treatment group firm in the year when the Provisions are implemented. | Choice |
| **CN** | Herfindahl–Hirschman Index (HHI) of the proportion of a firm’s pledged shares to the total shares for each creditor. ${CN}_{i,t}=\sum_{j=1}^{n} S_{ij}^{2}$ | Choice |
| ***Control variables*** |  |  |
| **Size** | Logarithm of asset value. | CSMAR |
| **Lev** | Ratio of total liability to total assets. | CSMAR |
| **Growth** | Growth rate of operating income. | CSMAR |
| **CFO** | Net cash flow from operating activities divided by total assets. | CSMAR |
| **Div_Yield** | Common dividend divided by market value of common stock. | CSMAR |
| **Big4** | Equal to 1 if the auditor is from one of the Big Four auditing firms, and 0 otherwise. | CSMAR |
| **MB** | Market-to-book ratio. | CSMAR |
| **Litigation** | Equal to 1 if the company has been sued, and 0 otherwise. | CNRDS |
| **SOE** | Equal to 1 if the firm is state-owned, and 0 otherwise. | CSMAR |
| **Firm_Age** | Age of the listed company. | CSMAR |
| **Capex** | Capital expenditures divided by total assets. | CSMAR |
| **Outside_Dir** | Fraction of independent directors. | CSMAR |
| **Board_Size** | Board size. | CSMAR |
| **Mas** | The percentage of a firm’s shares held by the CEO at the end of the fiscal year. | CSMAR |
